# Coexistence of primary thyroid diffuse large B cell lymphoma and papillary thyroid carcinoma in a patient with Hashimoto’s thyroiditis: a case report and literature review

**DOI:** 10.3389/fonc.2023.1248830

**Published:** 2023-10-05

**Authors:** Juncheng Li, Shengdong He, Juan Xu, Gang Xue

**Affiliations:** Department of Thyroid and Breast Surgery, General Hospital of Western Theater Command of Chinese People’s Liberation Army, Chengdu, China

**Keywords:** primary thyroid lymphoma, papillary thyroid carcinoma, coexistence, diffuse large B-cell lymphoma, case report

## Abstract

Papillary thyroid carcinoma (PTC) is the most common pathological type of thyroid malignancy and also has an excellent prognosis. Primary thyroid lymphoma (PTL) is rare and has a poor prognosis. The co-occurrence of both malignancies is extremely rare, and the preoperative diagnosis is rather difficult. We report the case of a patient with both PTC and PTL in the setting of Hashimoto’s thyroiditis (HT). A 59-year-old female patient was referred to our department for progressive enlargement of the thyroid gland over a few months. The imaging results demonstrated an enlarged thyroid and a mass in the thyroid. Total thyroidectomy and bilateral central neck node dissection were conducted. The final diagnosis of the coexistence of thyroid diffuse large B cell lymphoma and PTC was confirmed by histopathology and immunohistochemistry. The patient received radiation therapy and six cycles of chemotherapy combined with targeted therapy, including rituximab, cyclophosphamide, doxorubicin, vindesine, and prednisone (R-CHOP). After 6 months of follow-up, neither tumor has recurred. It is important for physicians to keep PTL in mind for differential diagnosis in HT patients with sudden thyroid enlargement.

## 1 Introduction

Papillary thyroid carcinoma (PTC) accounts for approximately 85%–90% of all cases of thyroid malignancies, and its incidence has risen considerably across the world in the past few decades (1). It occurs most predominantly in women and has an excellent prognosis, with a 10-year survival >91% and 15-year survival >87% (2, 3). Conversely, primary thyroid lymphoma (PTL) accounts for 5% of thyroid malignancies and 2% of extranodal lymphomas, with an estimated annual incidence of two per 1 million (4, 5). The most common subtype of PTL is diffuse large B cell lymphoma (DLBCL), accounting for more than 50% of cases (6). It occurs most predominantly in women between 60 and 69 years old (7). DLBCL usually presents with a more aggressive course and has a worse outcome, with the 5-year disease-specific survival rate being 75% (8). In recent decades, the incidence of Hashimoto’s thyroiditis (HT) has also significantly increased (9). Currently, it is believed that HT is a “double-edged sword” in PTC patients. It increases the risk of PTC but is a protective factor against PTC progression (10). HT is also known to increase the relative risk of developing PTL; nevertheless, only 0.5% of all HT cases develop PTL (11).

The concomitance of PTC and PTL in the same patient is extremely rare. The present case report aims to describe the coexistence of PTC with DLBCL in a background of HT for providing reference for future clinical practice.

## 2 Case presentation

A 59-year-old female patient was referred to our department for progressive enlargement of the thyroid gland over a few months. The patient denied dysphagia, hoarseness, shortness of breath, weight loss, fever, or night sweats. The patient had no significant history of past illnesses and had no family history of cancer. She denied any history of thyroid disease or radiation exposure. On physical examination, her thyroid gland was enlarged, with the right lobe being larger than the left; however, there was no pain or tenderness. There was an approximately 5 cm × 4 cm palpable mass in the right lobe and approximately 1 cm × 1 cm palpable nodule in the isthmus lobe. There were no palpable cervical lymph nodes. The rest of the findings of the physical examination were unremarkable.

**Figure 1:**
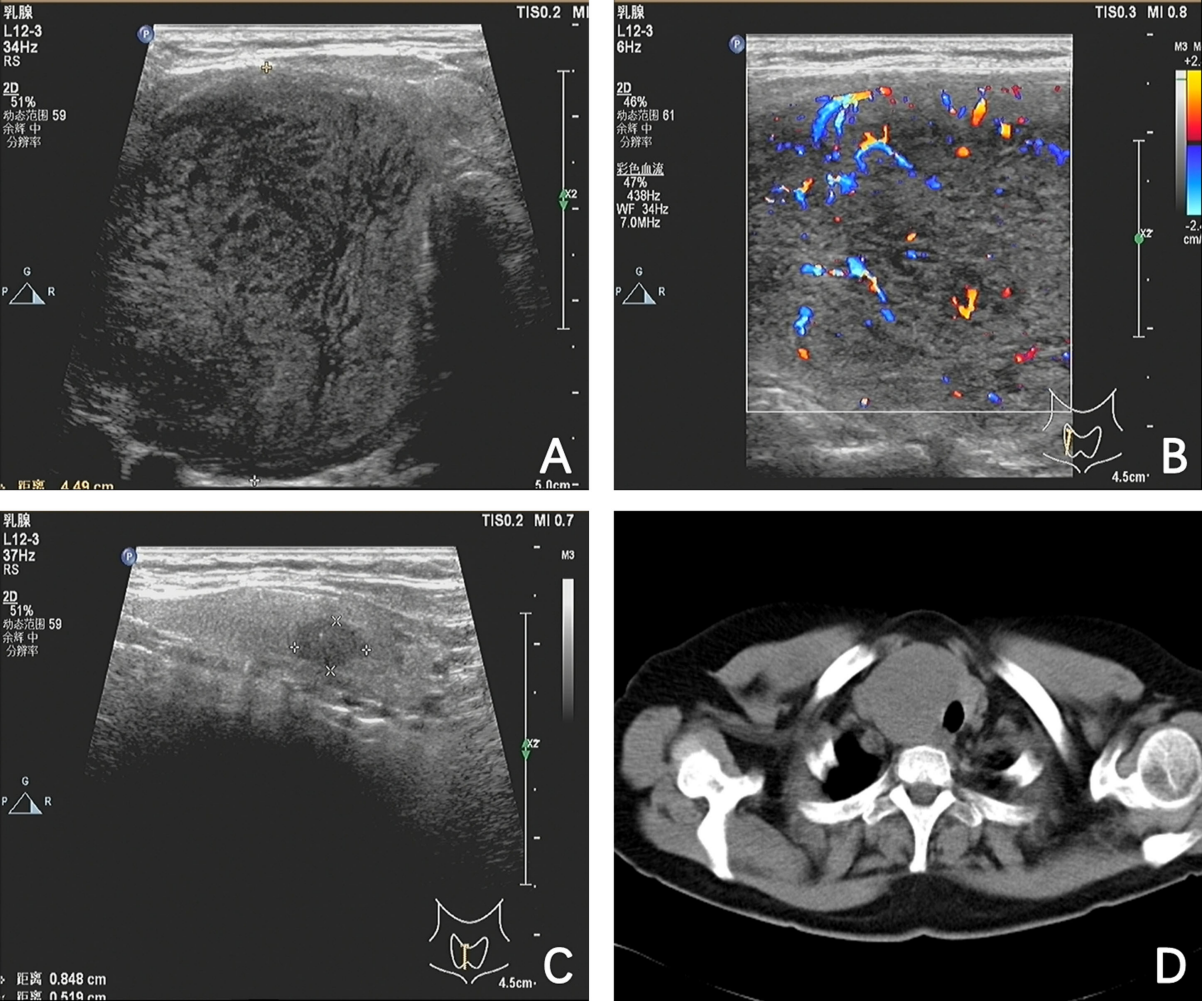
Ultrasound and computed tomography (CT) images of a tumor. **(A)** A hypoechoic mass measuring 65.4 mm × 34.1 mm × 57.5 mm in the right lobe. **(B)** The hypoechoic mass with rich blood flow signals in the right lobe. **(C)** A hypoechoic nodule with microcalcifications in the isthmus lobe. **(D)** A CT scan of the neck revealed an uneven thyroid gland with a slightly low-density mass in the isthmus and right lobe, ranging approximately 5.3 cm × 4.3 cm.

The routine laboratory tests of thyroid function were normal, although the serum antithyroid peroxidase antibody level was significantly elevated. The thyroid function tests were as follows: free T4, 1.19 ng/dL (reference range, 0.90 to 1.76); T3, 1.33 ng/mL (reference range, 0.58 to 1.82); thyroid stimulating hormone (TSH), 3.074 mIU/L (reference range, 0.550 to 4.780); thyroglobulin level, 20.50 ng/mL (reference range, 1.59 to 50.03); and antithyroglobulin antibody level, <15.00 U/mL (reference range, 0 to 60), which were within normal limits, but the antithyroid peroxidase antibodies were significantly elevated (>1,300 U/mL; reference range, 0 to 60). The tumor marker such as CEA was reported in the normal range. Thyroid ultrasonography revealed diffuse enlargement of a thyroid gland, with heterogeneous background parenchyma. In particular, two hypoechoic nodules were detected: one in the right lobe, measuring 65.4 mm × 34.1 mm × 57.5 mm, and the other in the isthmus lobe, measuring 8.5 mm × 5.2 mm × 5.7 mm (**Figures 1A–C**). The result of an ultrasound (US) examination revealed that the nodule in the isthmus lobe was suspicious of malignancy and the nodule in the right lobe was greater than 4 cm. In view of these, the patient was admitted to our department for surgery. A preoperative computed tomography (CT) scan of the neck revealed an uneven thyroid gland with a slightly low-density mass in the isthmus and right lobe, ranging approximately 5.3 cm × 4.3cm (**Figure 1D**).

**Figure 2:**
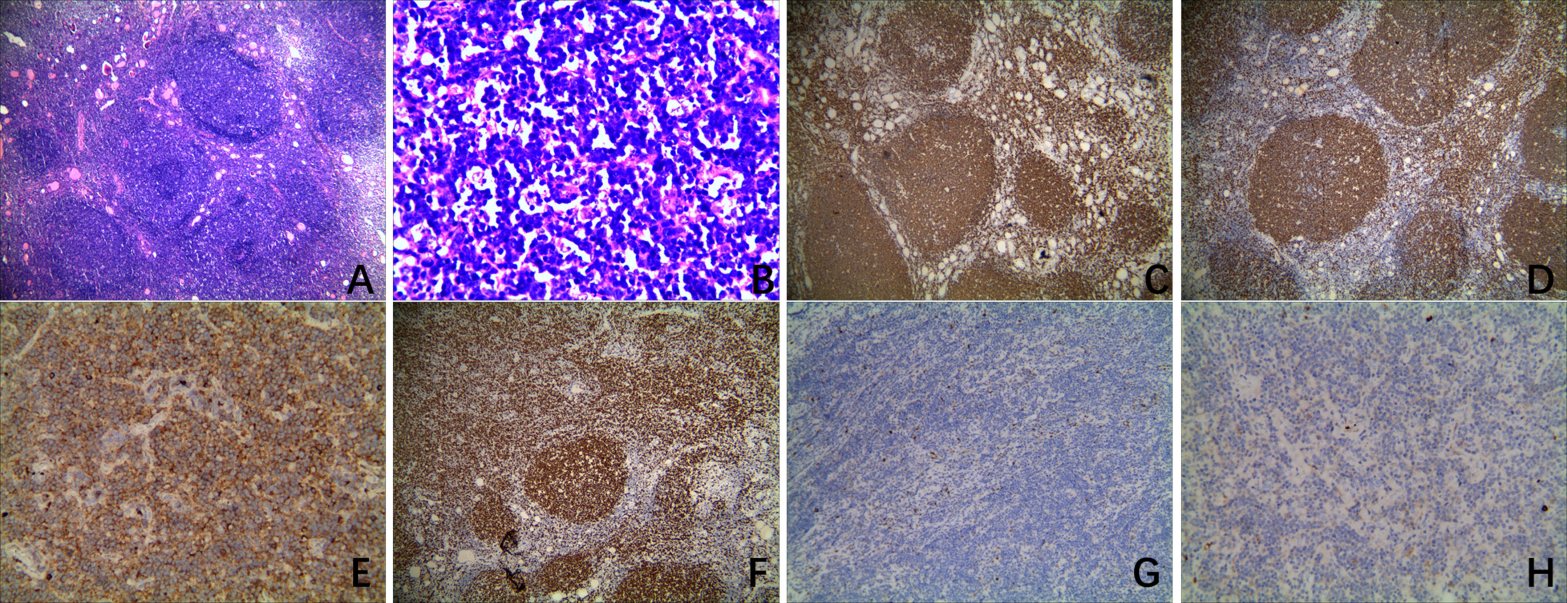
Histologic and immunohistochemical images of a primary thyroid lymphoma in the right lobe. **(A)** Microscopically, a histologic analysis of hematoxylin and eosin staining (×4) revealed that much of the normal thyroid architecture was replaced by dense, diffuse infiltrates of large atypical lymphocytes. Several scattered reactive germinal centers were also observed in the histologic sections. **(B)** Histologic analysis of hematoxylin and eosin staining (×40) revealed that the lymphomas were composed of a monotonous population of large atypical lymphocytes with irregular nuclei, condensed chromatin, and small nucleoli. **(C–H)** Immunohistochemical staining showing positive expressions for CD20 (×4), PAX-5 (×4), Ki-67 (×4), and Kappa (×20) and negative expressions for CyclinD1 (×10) and Lambda (×20).

The patient in our case refused to undergo ultrasound-guided fine needle aspiration biopsy and requested a surgical biopsy. After intraoperative frozen pathology detected that the isthmus nodule was papillary thyroid carcinoma (PTC) and the mass in the right lobe was a lymphoepithelial lesion, a total thyroidectomy and bilateral central neck node dissection was performed. Both the parathyroid hormone (PTH) level and the serum calcium level were measured on the first morning after surgery. The PTH level was 29.30 pg/ml (reference range, 12 to 88), and the serum calcium level was 2.12 mmol/L (reference range, 2.00 to 2.70). There were no hoarseness and dysdipsia after surgery. The histopathological examination disclosed a 0.9 cm × 0.6 cm papillary carcinoma in the isthmus thyroid gland, accompanied with diffuse large B-cell lymphoma (DLBCL) in the right lobe. No metastatic lymph nodes were found. Microscopically, background thyroid tissue showed diffuse lymphocyte infiltration with germinal center formation, follicles of various sizes with scanty colloid, and the presence of Hürthle cells, which are all characteristics of HT. The histological analysis of the isthmus nodule demonstrated that the tumor cell nuclei were enlarged and oval, with nuclear features such as powdery chromatin, nuclear grooves, and small nucleoli, which supported the presence of a PTC. The hematoxylin and eosin (H&E) staining of the right lobectomy sample revealed that much of the normal thyroid architecture was replaced by dense, diffuse infiltrates of large atypical lymphocytes with irregular nuclei, condensed chromatin, and small nucleoli (**Figures 2A, B**). Moreover several scattered reactive germinal centers were seen (**Figure 2A**). The immunohistochemistry (IHC) showed CD20 (+), CD19 (+), CD22 (+), CD3 (-), CD5 (-), CD30 (-), CD10 (-), CD23 (-), CD21 (-), Bcl-6 (+), Bcl-2 (-), MUM-1 (-), C-myc (+, 5~10%), TdT (-), CyclinD1 (-), PAX-5(+), PD-1 (-), P53 (+, 40%–50%), Ki-67 (+, 60%–70%), Kappa (+), and Lambda (-) (**Figures 2C–H**). The *in situ* hybridization of EBER 1/2 was negative. The fluorescence *in situ* hybridization detection indicated that c-myc, bcl-2 and bcl-6 gene translocations, and 1p36 gene deletion were negative. Additionally, the gene rearrangement assay (PCR+GENESCAN) suggested that an IgK clonal amplification peak rather than an IgH clonal amplification peak was detected. Based on morphology, immunophenotype, and molecular detection results, the diagnosis of DLBCL [germinal center B-cell lineage (GCB type, Hans classification)] was clear. Postoperative staging procedures for DLBCL were performed, including total body CT, gastrointestinal endoscopy, and bone marrow biopsy. There was no evidence of a systemic disease or metastases, and there were no systemic B symptoms related to lymphoma, corresponding to stage IE according to Ann Arbor classification.

The patient received radiation therapy and six cycles of chemotherapy combined with targeted therapy for DLBCL, including rituximab, cyclophosphamide, doxorubicin, vindesine, and prednisone (R-CHOP). Meanwhile, she is currently taking levothyroxine at 75 ug per day for TSH suppressive therapy. At 6 months after surgery, a thyroid function test was performed, and the antithyroglobulin antibody levels and thyroglobulin levels were measured [free T4, 1.27 ng/dL (reference range, 0.90 to 1.76); T3, 1.03 ng/mL (reference range, 0.58 to 1.82); TSH, 0.340 mIU/L (reference range, 0.550 to 4.780); antithyroglobulin antibody, 5.4 IU/mL (reference range, 0 to 4.5); thyroglobulin, 0.01 ng/mL (reference range, 1.59 to 50.03)]. The patient have good adherence to treatment and follow-up, and neither tumor has recurred during the 6-month period.

## 3 Discussion

The rapid increase in the detection rate of thyroid malignancy in the past decade has been facilitated by the improved resolution of high-frequency US and greater prevalence of physical examination. PTC is the most common pathological type of thyroid malignancy and also has an excellent prognosis. However, PTL is rare and has a poor prognosis. The PTL cases are typically non-Hodgkin’s lymphoma of B cell origin, which occur most frequently in elderly women linked with chronic autoimmune thyroiditis. The most common clinical manifestation is a rapidly growing neck mass and is reported in 88% of cases, most commonly in those with DLBCL (8, 12). To the best of our knowledge, the coexistence of thyroid DLBCL and PTC is very rare, and only a handful of cases have been previously reported in the literature (13–18). A comparison of findings between the current and previously reported cases is depicted in **Table 1**.

**Table 1:** Summarization of cases of coexisting papillary thyroid carcinoma and primary thyroid diffuse large B-cell lymphoma.

Case ID	Sex/age (years)	Clinical symptom	Thyroid ultrasound	Thyroid function	HT	FNA	Final pathologic diagnosis	Stage of thyroid DLBCL	Treatment	Follow-up	Reference
1	F/50	Dyspnea, difficulty swallowing, and a rapidly growing mass in the neck	A 6 cm × 3 cm nodule in the right lobe	Normal	Positive	Suspicious for lymphoma	Primary thyroid DLBCL in the right lobe, and PTC in the left lobe	IE	Total thyroidectomy and levothyroxine	Alive and well, 2 years	(13)
2	M/41	Fast painless thyroid enlargement and a cervical mass	A 7.7 cm× 3.6 cm × 2.9 cm heterogeneous hypoechoic nodularity and diffused hyperechoic areas in the left lobe	Normal	Positive	No	Primary thyroid DLBCL coexistent with PTC in the left lobe	IVE	Resection of the isthmus and left lobe of the thyroid, dissection of the left cervical lymph nodes, and CHOP chemotherapy	Alive and well, 2 months	(14)
3	F/66	A fast, painless enlargement in the right anterior side of the neck and dysphagia	A 3.2 cm × 2.0 cm hypoechoic nodule in the right lobe	Unknown	Positive	Atypical epithelial cells and lymphocytic infiltration	Primary thyroid DLBCL coexistent with PTC in the right lobe	IE	Total thyroidectomy and chemotherapy	Alive and well, 2 years	(15)
4	F/77	A progressively enlarging thyroid gland	Diffuse bilobate enlargement of the thyroid with a poorly defined boundary and heterogeneous nodularity	Unknown	Positive	A prominent population of monotonous non-cohesive large cells	Primary thyroid DLBCL coexistent with PTC in the right lobe	IE	Total thyroidectomy and CHOP chemotherapy	Alive and well, 2 years	(16)
5	F/37	Dyspnea and swallowing difficulties caused by a rapidly expanding mass in the neck	Diffuse homogeneous enlargement of the right thyroid lobe to 3.2 cm in diameter	Unknown	Positive	Unknown	Primary thyroid DLBCL complicated with PTC in the right lobe	IE	Lobectomy of the right thyroid gland and CHOP chemotherapy	Alive and well, 1 year	(17)
6	F/52	Goiter and progressively increasing dificulty in breathing	Heterogeneously enlarged thyroid gland with left-sided cervical lymphadenopathy	Normal	Positive	Scattered lymphocytes	Primary thyroid DLBCL in the left lobe and PTC in the right lobe	IE	Total thyroidectomy with central, left level II to V neck dissection, thyroxine therapy, and R-CHOP chemotherapy	Alive and well, 1 year	(18)
7	F/59	Progressive enlargement of the thyroid gland	A 6.5 cm × 3.4 cm × 5.7 cm nodule in the right lobe and the other 8.5 mm × 5.2 mm × 5.7 mm nodule in the isthmus lobe	Normal	Positive	No	Primary thyroid DLBCL in the right lobe and PTC in the isthmus lobe	IE	Total thyroidectomy with bilateral central neck node dissection, thyroxine therapy, and R-CHOP chemotherapy	Alive and well, 6 months	Our case

F, female; M, male; HT, Hashimoto’s thyroiditis; FNA, fine needle aspiration; DLBCL, diffuse large B cell lymphoma; PTC, papillary thyroid carcinoma.

Autoimmune thyroid diseases, especially HT, are known to increase the relative risk of developing PTL and PTC. As summarized in **Table 1**, all of these reported cases had a history of HT. There are three possible pathogenic mechanisms. Firstly, the chronic inflammatory reactivity creates a favorable environment for malignant transformation. Secondly, the increased level of TSH is an important stimulation factor for follicular epithelial hyperplasia, which promotes malignant transformation. Thirdly, RET/PTC gene rearrangement may be involved in the early stages of HT and tumor development (10). However, up to now, many critical steps of malignant transformation are still unknown.

Usually, US is the first-line diagnostic modality used in the work-up of thyroid enlargement and nodules. Nonetheless, it presents with a nonspecific appearance such as a diffusely enlarged thyroid gland accompanied with a node, which is also typical of severe HT and non-diagnostic for PTL. As shown in **Table 1**, all of these reported cases had no specific ultrasound findings. Tissue analysis is essential for establishing an accurate diagnosis. Accordingly, ultrasound-guided fine needle aspiration (FNA) is the next step for the diagnostic strategy. Recently, with the rapid development of molecular pathology, the accuracy of FNA has obviously improved. However, it was reported that this test has a low sensitivity of 48% in the diagnosis of PTL because of a high rate of false-negative results (19). No wonder that these patients in previously reported cases had hardly been correctly diagnosed by FNA cytology. In our case, we did not perform ultrasound-guided FNA on thyroid nodules preoperatively. The reasons were as follows: Ultrasound-guided FNA has a low sensitivity in the diagnosis of PTL. Moreover, the patient in our case refused to have this examination conducted and requested a surgical biopsy. Meanwhile, there were indications for surgical biopsy, such as the nodule in the isthmus lobe being suspicious of malignancy and the nodule in the right lobe being greater than 4 cm. In view of these, we performed intraoperative frozen pathology instead of ultrasound-guided FNA in our case. Intraoperative frozen pathology can distinguish the nature of thyroid nodules and then determine the surgical procedure. Therefore, the reoperation of cervical lymph node dissection has fallen off considerably.

Surgical open biopsy and IHC are recognized as the gold standard for PTL diagnosis when the previous tests have not been conclusive. Thyroid DLBCL often appears histopathologically as a relatively uniform population of large, abnormal lymphoid cells with lymphoepithelial foci and decreased or absent colloid (8). On immunohistochemical staining, the positivity of CD19, CD20, CD22, and PAX-5 identifies a B cell lineage to the lymphoid cells. The positivity of CD10 can help to identify follicular lymphoma. While CyclinD1 is positive in most mantle cell lymphoma, it is typically negative in DLBCL. These antibodies, including CD 10, Bcl-6, and MUM-1, contribute to the identification of a DLBCL subtype. Meanwhile, the expression of Ki-67 is often more than 40%. If B-cell lymphomas are monoclonal, the expression of either lambda or kappa light chains will be restricted. On molecular pathology, the gene rearrangement assay and detection of c-myc, bcl-2, and bcl-6 gene translocations will contribute to the diagnosis of DLBCL and the prediction of a prognosis. In our case, as in a few other cases, the thyroid DLBCL was confirmed only after surgery. The presented case was positive for CD20, CD19, CD22, Bcl-6, PAX-5, Ki-67, and Kappa and negative for CD5, CD10, MUM-1, CyclinD1, and Lambda, which confirmed the final diagnosis DLBCL [germinal center B-cell lineage (GCB type, Hans classification)]. Based on the negativity of c-myc, bcl-2, and bcl-6 gene translocations, the diagnosis of double-hit lymphoma or triple-hit lymphoma was ruled out.

Regarding treatment strategies, experience in the optimal management of synchronous PTL and PTC is limited. The main treatment for PTC is surgical resection. Postsurgical therapies of PTC are thyroid-stimulating hormone suppression and radioiodine therapy, based on the extent of the disease at surgery, status of regional lymph nodes, age of the patient, and assigned risk group. The treatment of PTL depends on the histological subtype and staging based on the Ann Arbor Classification System (**Table 2**). Specifically for DLBCL, the National Comprehensive Cancer Network International Prognostic Index (NCCN-IPI) (**Table 2**) is a widely used scoring system to predict the prognosis. A recent study in 2022 showed that 87.6% of new PTL cases were Ann Arbor stages IE–IIE (20). The present case was staged as IE and categorized under NCCN-IPI as low risk. Surgery seems to play a limited role in PTL and is only really necessary in relieving compressive symptoms and obtaining specimens for final diagnosis. Nowadays, the combination of systemic chemotherapy and local–regional radiotherapy is the foundation of PTL treatment. Single chemotherapy or radiotherapy alone has been administered for localized and indolent lymphomas, while combination therapy is recommended for disseminated and aggressive lymphomas. Because of the aggressive nature and the propensity for systemic recurrence, radiochemotherapy is the best choice for DLBCL, which is the therapeutic strategy for the patient in this report. Thus, when PTC and PTL do coexist in the same patient, treatment strategies should be individualized, focused on whichever tumor is at the worse stage and condition, but an ideal strategy entails the optimal treatment of both tumors.

**Table 2:** Ann Arbor staging for primary thyroid lymphoma and National Comprehensive Cancer Network International Prognostic Index.

Ann Arbor staging for primary thyroid lymphoma	
Stage	Location of disease outside of the thyroid
IE	None
IIE	Regional lymph nodes
IIIE	Lymph nodes on both sides of the diaphragm
IVE	Systemic dissemination
National Comprehensive Cancer Network International Prognostic Index	
Risk factors	Score
Age, years	
>40 to ≤60	1
>60 to ≤75	2
>75	3
LDH, normalized	
>1 to ≤3	1
>3	2
Ann Arbor stage III– IV	1
Extranodal disease^a^	1
Performance status ≥2	1
Risk stratification	Score
Low	0–1
Low-intermediate	2–3
High-intermediate	4–5
High	6–8

a Disease in bone marrow, central nervous system, liver/gastrointestinal tract, or lung.

## 4 Conclusion

The coexistence of PTL and PTC is extremely rare, making its diagnosis, management, and treatment challenging. The diagnosis of PTL is based on histopathology and immunohistochemistry. Because of the high invasiveness and poor prognosis of DLBCL, early diagnosis is crucial to intervene promptly and achieve a promising outcome. Once diagnosed, the treatment has to prioritize the tumor with the worse stage and condition. As for DLBCL, multimodal therapy including radiotherapy and chemotherapy are highly recommended to prolong a patient’s survival.

## Data availability statement

The raw data supporting the conclusions of this article will be made available by the authors, without undue reservation.

## Ethics statement

The studies involving humans were approved by the ethics committee of the General Hospital of Western Theater Command. The studies were conducted in accordance with the local legislation and institutional requirements. The participants provided their written informed consent to participate in this study. Written informed consent was obtained from the individual(s) for the publication of any potentially identifiable images or data included in this article.

## Author contributions

JL, SH, and JX wrote the manuscript. GX reviewed the manuscript. All authors contributed to the article and approved the submitted version.
